# ﻿A contribution to the knowledge of cave-adapted ground beetles from Guiyang, central Guizhou Province, southwestern China (Coleoptera, Carabidae, Trechini)

**DOI:** 10.3897/zookeys.1075.73318

**Published:** 2021-12-07

**Authors:** Mingyi Tian, Guangyuan Cheng, Sunbin Huang

**Affiliations:** 1 Department of Entomology, College of Plant Protection, South China Agricultural University, 483 Wushan Road, Guangzhou, 510642, China; 2 Haixia Caving, Bureau of Ecology and Environment, no. 7 Building, Financial City, Guanshanhu, Guiyang, Guizhou, 550081, China; 3 Mécanismes adaptatifs et évolution (MECADEV), UMR 7179 CNRS–MNHN, Muséum national d’Histoire naturelle, CP50, 57 Rue Cuvier, F–75005 Paris, France

**Keywords:** Hypogean, new genus, new species, semi-aphaenopsian, trechines

## Abstract

A new genus and two new species of cavernicolous trechines are reported from central Guizhou Province, southwestern China. *Haixiaphaenops***gen. nov.** is established to place a new species discovered in two limestone caves in northern Qingzhen Shi: *H.jinxiaohongae***sp. nov.** (Dawan Dong cave and Changtu Dong and Dawan Dong caves). This new genus is allied to *Zhijinaphaenops* Uéno & Ran, 2002. *Zhijinaphaenopszhaofeii***sp. nov.** is described from Zhangkou Dong cave in northern Jiuzhuang Zhen of Xifeng County. In addition, two new localities of the species *Zhijinaphaenopsjingliae* Deuve & Tian, 2015, and two new localities of *Sinaphaenopschengguangyuani*Ma et al. 2020 are provided. A distribution map for all cavernicolous trechine beetles known in Guiyang is provided.

## ﻿Introduction

Undoubtedly, Guizhou is the province harbouring the richest cave specific diversity in China in terms of hypogean trechine beetles (Tian et al. 2016, 2020; Chen et al. 2017; Huang et al. 2020; Tian et al. 2021). Of the 168 cave species of trechines known in China, 62 are occurring in Guizhou Province. But the subterranean fauna of ground beetles is poorly studied in Guiyang Shi, central Guizhou. Only three species in two genera of cave-adapted trechines have been reported from this area. The genus *Zhijinaphaenops* Uéno & Ran, 2002 was formerly described from Zhijin County, Bijie Shi, central Guizhou, then reported from several other counties, viz. Dafang, Xifeng, Zunyi, and Weng’an. Nine species are included in the genus so far (Uéno and Ran 2002; Deuve and Tian 2015, 2018). Among them, two species were recorded from Xifeng County, Guiyang Shi: *Zhijinaphaenopsjingliae* Deuve & Tian, 2015 and *Z.liuae* Deuve & Tian, 2015.

*Sinaphaenops* Uéno & Wang, 1991 is a large genus, composed of 13 species so far, ranging in southern Guizhou and also extending to Huanjiang County, northern most Guangxi (Uéno and Wang 1991; Uéno and Ran 1998; Uéno 2002; Deuve and Tian 2014; Chen et al. 2017; Ma et al. 2020). Amongst them, only *S.lipoi*Chen et al. 2020 is known from Guiyang (Chen et al. 2020).

**Figure 1.**
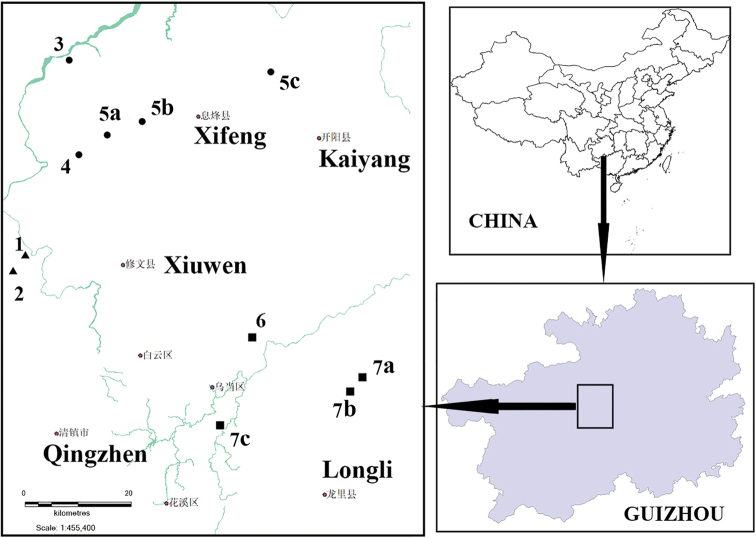
A distribution map of cavernicolous trechine beetles in Guiyang, central Guizhou Province. Triangles: *Haixiaphaenops* gen. nov.; dots: *Zhijinaphaenops*; squares: *Sinaphaenops***1***H.jinxiaohongae* sp. nov. / Changtu Dong **2***H.jinxiaohongae* sp. nov. / Dawan Dong **3***Z.zhaofeii* sp. nov. / Zhangkou Dong **4***Z.liuae* Deuve & Tian, 2015 / Hejia Dong **5a***Z.jingliae* Deuve & Tian, 2015 / Zhangkou Dong **5b***Z.jingliae* / Mafen Dong **5c***Z.jingliae* / Wenquan Dong **6***S.lipoi*Chen et al. 2020 / Da Dong **7a***S.chengguangyuani*Ma et al. 2020 / Shuijing Dong **7b***S.chengguangyuani* / Duocai Dong **7c***S.chengguangyuani* / Jianlong Dong.

Led by Guangyuan Cheng, the second author, the local cavers from the Haixia Caving (a cave exploration team in Guiyang) have begun to carry out biological surveys in recent years. They have visited many limestone caves in central Guizhou Province and discovered some interesting cavernicolous ground beetles (Fig. 1). For instance, *Sinaphaenopschengguangyuani*Ma et al. 2020 was formerly found by them in a limestone cave in Longli County of Qiannan Buyi & Maio Autonomous Prefecture, close to Guiyang (Ma et al. 2020). Two more localities, from Guiyang and Longli respectively, of this beautiful species are now confirmed. In Xifeng County, they discovered two other caves where *Zhijinaphaenopsjingliae* is living. In Qingzhen Shi, they found two beetle individuals in the cave Dawan Dong and another beetle in the cave Changtu Dong. These beetles are members of an undescribed species belonging to an unknown genus allied to *Zhijinaphaenops*.

Hence, we establish a new genus to accommodate the new species found in the caves Dawan Dong and Changtu Dong, describe a new *Zhijinaphaenops* species from the cave Zhangkou Dong in Xifeng County and provide new localities for *Zhijinaphaenopsjingliae* and *Sinaphaenopschengguangyuani* in the suburbs of Guiyang, the capital city of Guizhou Province.

## ﻿Materials and methods

The beetle specimens were collected in caves by hand or by using an aspirator, and kept in vials with 50% ethanol. One exemplar of each species was placed into 95% ethanol for DNA sequencing. Dissections and observations were made by using a Leica MZ75 dissecting stereomicroscope (Wetzlar, Germany). Dissected genitalia, including the median lobe and parameres of aedeagus, were glued on small transparent plastic cards and pinned under the specimen from which they were removed. Digital pictures were taken using a Canon EOS 5D Mark III camera (Tokyo, Japan), and then processed by means of Adobe Photoshop CS5 software (Adobe System Incorporated, California, USA).

### ﻿Measurements and terminologies used in the text follow Tian et al. (2016). The abbreviations used in the text are as follow:

**HLm** length of head including mandibles, from apex of right mandible to occipital suture;

**HLl** length of head excluding mandibles, from front of labrum to occipital suture;

**HW** maximum width of head;

**PrL** length of prothorax, along the median line;

**PnL** length of pronotum, as above;

**PrW** maximum width of prothorax;

**PnW** maximum width of pronotum;

**PfW** width of pronotum at front;

**PbW** width of pronotum at base;

**EL** length of elytra, from base of scutellum to elytral apex;

**EW** maximum width of combined elytra.

All material is deposited in the insects collection of South China Agricultural University, Guangzhou, China (SCAU).

## ﻿Taxonomy

### Tribe Trechini Bonelli, 1810

#### ﻿ Haixiaphaenops gen. nov.

Taxon classificationAnimaliaColeopteraCarabidae

84C9696D-E48F-5BED-A91B-58EDE23D6F49

http://zoobank.org/7BBA8EF9-21D8-460D-A361-CC134DF019CD

##### Type species:

*Haixiaphaenopsjinxiaohongae* sp. nov. (caves Dawan Dong and Changtu Dong, Qingzhen, Guiyang)

##### Generic characteristics.

Medium-sized cave trechine, depigmented and eyeless, semi-aphaenopsian; body stout though fore body elongated, with moderately elongated appendages. Head longer than wide, 2 pairs of supraorbital setiferous pores present; frontal furrows rather long, incomplete, parallel-sided in most part, divergent posteriad; mandibles thin and very elongated, moderately hooked apically, right mandibular tooth bidentate though with 2 additional tiny denticles medially; labial suture absent; mentum 2-setose, base widely concave, submentum 10-setose; antennae thin and rather long, extending to apical 1/3 to 1/4 of elytra. Prothorax strongly convex and propleura notably visible from above; pronotum, much longer than wide, subparallel sided, disc covered with long setae, presence of only anterior lateral setae. Elytra elongated ovate, much wider than fore body; widest before middle, without humeral angles; base bordered, lateral margins well-bordered and ciliate throughout; disc extremely convex and tumid, partly concealing lateral margins; striae noticeable though punctures faint, intervals slightly convex; 3 discal setiferous pores present on each elytron, the preapical pores absent; the humeral group of the marginal umbilicate pores not aggregated, 1^st^ pore inwardly and backwardly shifted, 5^th^ and 6^th^ pores (middle group) moderately spaced. 1^st^ protarsomere dilated and elongated in male, inwardly spurred at apex; abdominal ventrite VII 6-setose in male.

##### Remarks.

*Haixiaphaenops* gen. nov. is allied to the genus *Zhijinaphaenops* Uéno & Ran, 2002 by sharing the following characteristics: (1) mentum and submentum completely fused; (2) prothorax strongly dilated and propleura notably visible from above, pronotum with only anterior latero-marginal setae; (3) only protarsomere 1 modified in male, which is long and inwardly spurred at apex; (4) pronotum covered with long setae; (5) elytra shortly pubescent, with hardly distinguishable striae, and the 1^st^ marginal umbilicate pore inwardly and backwardly shifted, located behind the level of the 3^rd^ pore. However, *Haixiaphaenops* gen. nov. readily differs from *Zhijinaphaenops* in several generic-level characters, such as: (1) 2 pairs of frontal pores present in *Haixiaphaenops* gen. nov., versus only the posterior pores present in *Zhijinaphaenops*; (2) antennae much shorter in *Haixiaphaenops* gen. nov., only extending at most to apical 1/4 of elytra, versus longer, projection over apices of elytra in *Zhijinaphaenops*; (3) pronotum elongated quadrate, nearly parallel-sided in *Haixiaphaenops* gen. nov., versus subcordate, not parallel-sided in *Zhijinaphaenops*; (4) elytra much stouter and more convex in *Haixiaphaenops* gen. nov., partly concealing lateral margins in median portion, versus more elongated and less convex in *Zhijinaphaenops*, with whole lateral margins visible from above; (5) base of elytra bordered in *Haixiaphaenops* gen. nov., versus unbordered in *Zhijinaphaenops*; and (6) male genitalia are small and stout, slightly bent medially, and widely rounded at apex in *Haixiaphaenops* gen. nov., versus large and slender, strongly arcuate medially, and more or less sharpened at apex in *Zhijinaphaenops*.

##### Etymology.

“Haixia” + “Aphaenops”, dedicated to Haixia Caving, a cave exploration team in Guiyang. Gender masculine.

##### Range.

China (Guizhou). Only one species of the genus was found in the limestone caves Dawan Dong and Changtu Dong in Qingzhen, northern Guiyang Shi (Fig. 1).

#### ﻿ Haixiaphaenops jinxiaohongae sp. nov.

Taxon classificationAnimaliaColeopteraCarabidae

**Figure 2.**
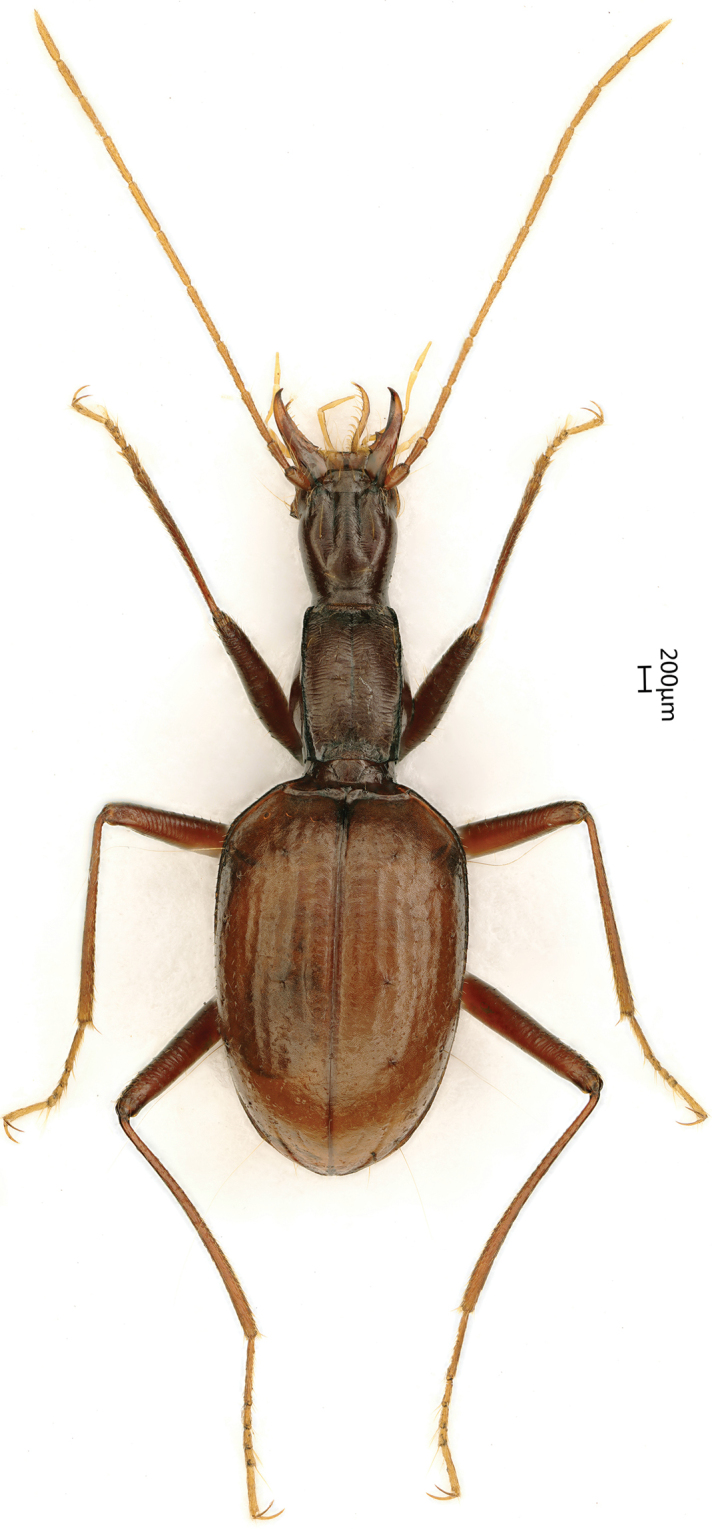
Habitus of *Haixiaphaenopsjinxiaohongae* gen. nov., sp. nov., holotype, male

**Figure 3.**
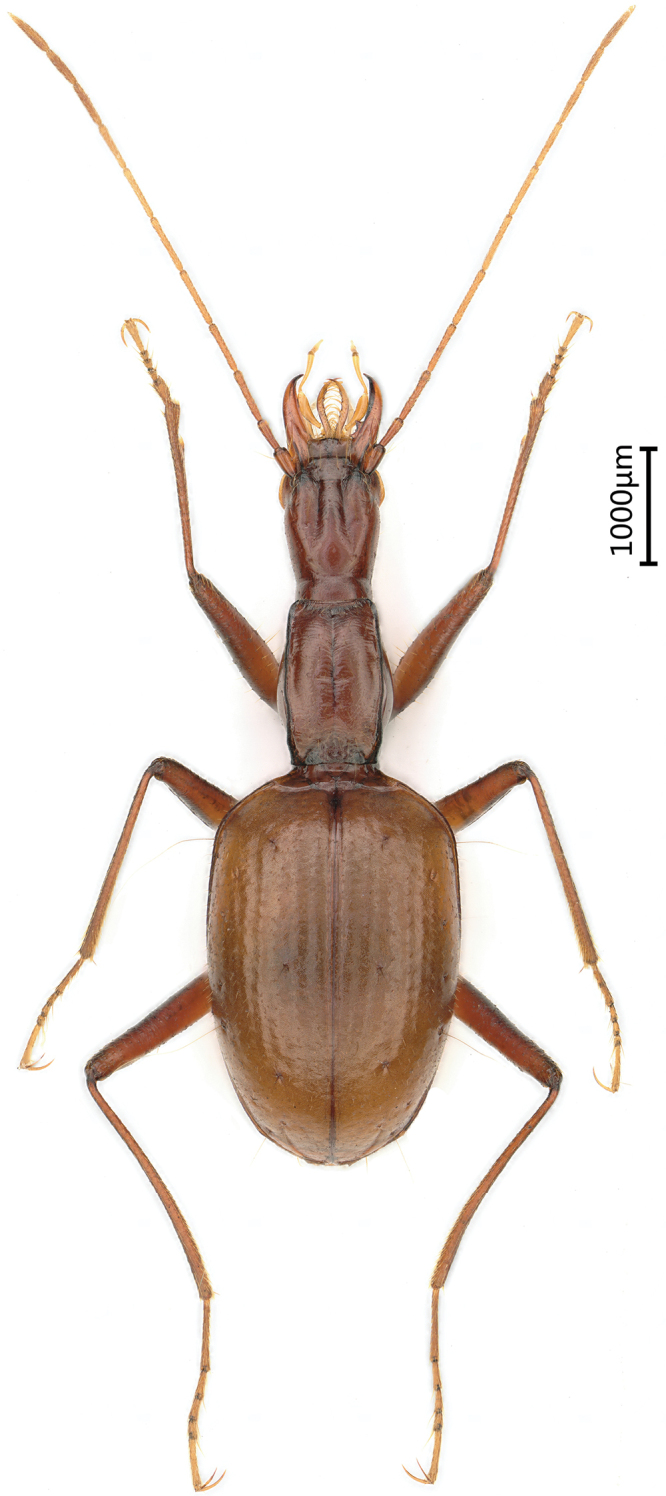
Habitus of *Haixiaphaenopsjinxiaohongae* gen. nov., sp. nov., paratype male, from the Cave Changtu Dong

**Figure 4.**
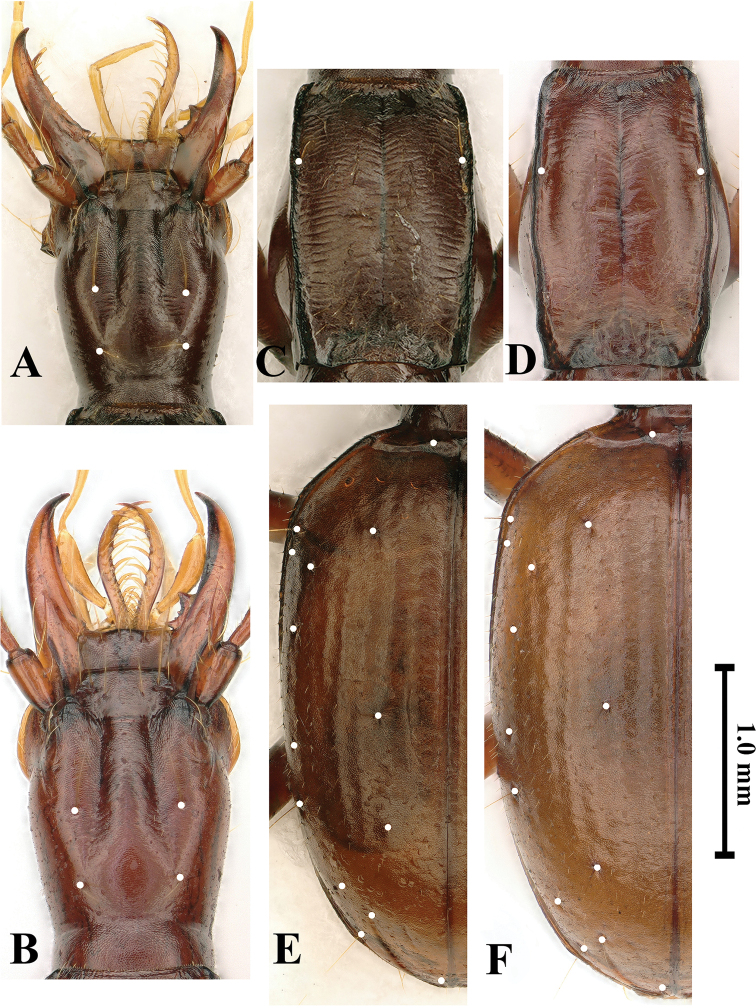
Shapes and chaetotaxy of head, pronotum and elytra of *Haixiaphaenopsjinxiaohongae* gen. nov., sp. nov. **A, C, E** holotype from Dawan Dong **B, D, F** paratype from Changtu Dong **A, B** head **C, D** pronotum **E, F** elytra. Scale bar: 1.0 mm (**A–F**).

**Figure 5.**
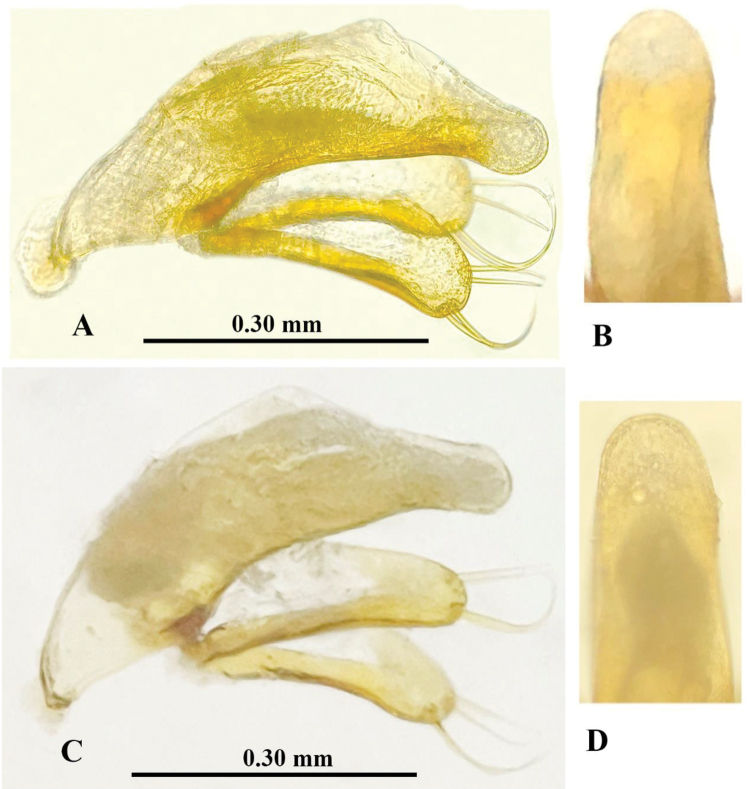
Male genitalia of *Haixiaphaenopsjinxiaohongae* gen. nov., sp. nov. **A, B** holotype from Dawan Dong **C, D** paratype from Changtu Dong.

**Figure 6.**
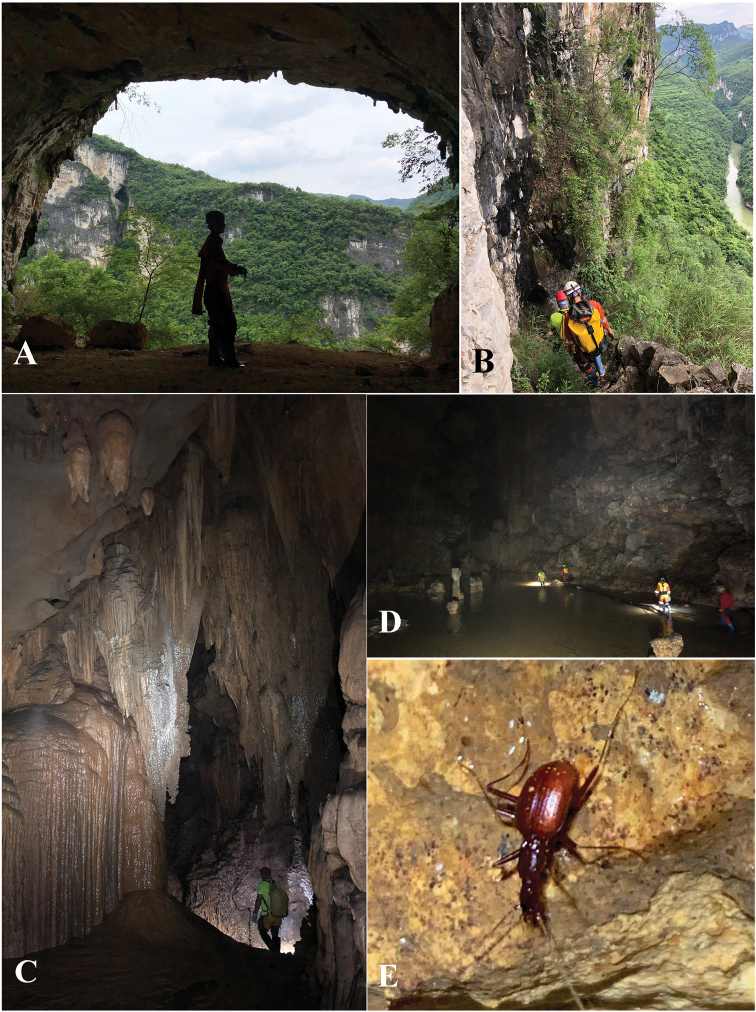
Dawan Dong cave, the type locality of *Haixiaphaenopsjinxiaohongae* gen. nov., sp. nov. **A, B** Entrances and environs **C, D** scenes inside the cave **E** a running individual of *H.jinxiaohongae* sp. nov. in the cave, courtesy of Mr Chenggang Wang.

**Figure 7.**
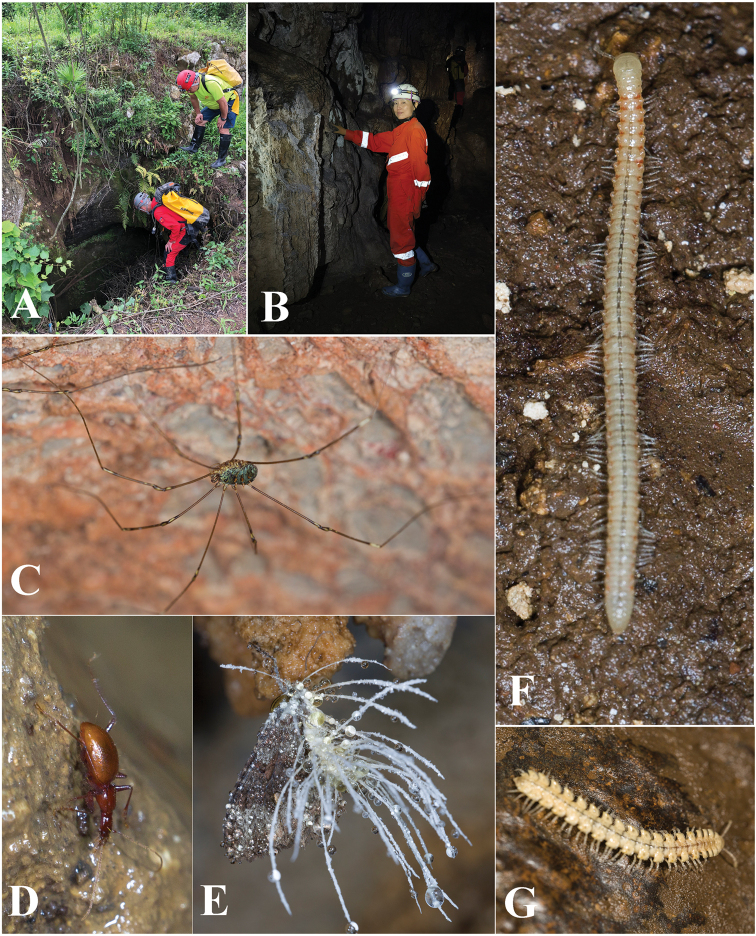
Changtu Dong cave, another locality of *Haixiaphaenopsjinxiaohongae* gen. nov., sp. nov. **A** one of the entrances **B** Xiaohong Jin is collecting in the cave **C** a harvestman **D** a running individual of *H.jinxiaohongae* gen. nov., sp. nov. **E***Cordyceps* fungus growing on a *Triphosa* moth **F** millipede *Glyphiulus* sp. **G** millipede *Pacidesmus* sp.

49191D89-8E38-5F63-B8B7-2A7830DEBDFD

http://zoobank.org/701ACD12-A45D-4794-9994-BF58B3BE90D4 (Chinese name: 晓红盲步甲) Figures 2 , 3 , 4 , 5 , 6 , 7

##### Type material.

***Holotype*** male: Guizhou, Qingzhen, Anliu, Yangtianwo, Dawan Dong cave (贵州省清镇市暗流镇大湾洞), 26°52'N, 106°24'E, 941 m, 2020–IV–19, leg. Xiaohong Jin & Guangyuan Cheng, in SCAU; ***Paratypes***: 1 male, same cave as holotype, 2021–VI–23, leg. Chenggang Wang; 1 male, Guizhou, Qingzhen, Anliu, Ximi, Changtu Dong cave (贵州省清镇市暗流镇长土洞), 26°51‘N, 106°21‘E, 1249 m, 2020–VI–27, leg. Chenggang Wang, Xiaohong Jing, Guangyuan Cheng, Yi Zhao & Mingyi Tian, both in SCAU.

##### Description.

Length: 6.5–6.8 mm (including mandibles); width: 1.0–1.1 mm. Habitus as in Figures 2, 3.

Head and pronotum dark brown (in holotype) or brown (in paratypes), elytra, femora and tibiae lighter, antennae, palps, and tarsi yellow. Head glabrous on upper surface, pronotum covered with long setae, elytra with short pubescence, ventral head covered with several setae; prosternum, meso- and metasterna and fore coxae glabrous, abdominal ventrites with dense and short pubescence, in particular on median portion. Microsculptural engraved meshes strongly transverse on head and pronotum, irregular isodiametric on elytra. Body moderately elongated, fore body (including mandibles) shorter than elytra.

Head (Fig. 4A, B) moderately elongated, distinctly longer than wide, HLm/HW= 2.27, HLl/HW= 1.56; widest at about middle of head from labrum; frons and vertex moderately convex, genae gently expanded anteriad (but hardly expanded in paratype specimens); frontal furrows nearly parallel-sided in anterior 2/3, then strongly divergent posteriad, ending before posterior supraorbital pores; clypeus transverse, 6-setose, with an additional short seta medially; labrum transverse, nearly straight at front margin, 6-setose; mandibles thin, gently unciform at apex; labial suture absent; mentum 2-setose on each side of tooth at base, mentum base widely concave; labial tooth short, thin and bifid at tip, about half length of lateral lobe; submentum 10-setose; ligula bisetose, paraglossae pubescent; palpomeres moderately elongate, all glabrous except 2^nd^ labial palpomere which is 4-setose (2 on inner margin and other 2 on outer margin at middle); the 2^nd^ labial palpomere 1.3 times as long as 3^rd^; 3^rd^ maxillary palpomere 1.2 times longer than 4^th^; suborbital pores near neck constriction; antennae slender and filiform, extending at about apical 1/4 of elytra, densely pubescent from pedicle to 11^th^ antennomere; scape thick, fusiform, smooth but with several rather long setae, slightly shorter than pedicle; 5^th^ and 6^th^ antennomeres longest; antennal ratio (relative length of each antennomere compared with scape in the holotype) as follows: 1^st^ (1.0), 2^nd^ (1.25), 3^rd^ (2.0), 4^th^ (2.0), 5^th^ (2.25), 6^th^ (2.25), 7^th^ (2.0), 8^th^ (2.75), 9^th^ (1.85), 10^th^ (1.5) and 11^th^ (2.0).

Prothorax (Fig. 4C, D) longer than wide, PrL/PrW = 1.25–1.45; distinctly wider than pronotum, PrW/PnW = 1.14–1.23; much shorter than head including mandibles, PrL/ HLm = 0.6–0.70, or as long as head excluding mandibles; propleura tumid, widest at about 2/5 from base. Pronotum as wide as head; much longer than wide, PnL/PnW=1.51–1.64, widest at about middle, sides nearly parallel-sided; lateral margins distinctly reflexed throughout, front angles obtusely rectangular, hind angles sharply angulate; anterior lateral setae at about 2/7 from front margin, posterior setae absent; both base and front unbordered, the former anterior margin is subrectilinear, basal margin emarginate medially, convex laterally; disc slightly convex, surface with short and transversal striations; basal foveae large and deep, reverse triangular in shape. Scutellum short and wide.

Elytra (Fig. 4E, F) ovate and stout, much wider than prothorax, EW/PrW = 2.10–2.18, much longer than wide, EL/EW = 1.52–1.60, widest at about basal 1/3 of elytra, extraordinarily convex, lateral margins of subapical portion invisible from above; humeri broadly rounded; lateral margins ciliate throughout; base bordered; striae easily traceable though punctures absent, apical striole well marked, ended at about apical 1/7 of elytra, in the joint area of 5^th^ and 6^th^ striae; intervals convex; chaetotaxy: basal pores present, along both sides of scutellum at apex; three discal pores present near position of 4^th^ stria, anterior and posterior pores at about 1/5 and 2/7 of elytra from base and from apex respectively, median pore near middle of elytra; humeral set of marginal umbilicate pores not aggregated, only 2^nd^ and 3^rd^ pores close to marginal gutter; 1^st^ pore transversely and backwardly shifted to site of 6^th^ interval, far behind level of 3^rd^ pore, making 4^th^ pore closer to 1^st^ than to 3^rd^; locations of middle set (5^th^ and 6^th^ pores) behind middle of elytra, not close to each other; umbilicate seta 8 clearly visible; angulo-apical pore present.

Legs quite stout, tibiae not longitudinally furrowed; protarsi short, 1^st^ protarsomere elongated and widened, denticulate on inner side of apex in male; 1^st^ tarsomere shorter than, subequal to, and longer than 2^nd^–4^th^ tarsomeres combined in pro-, meso- and metatarsi, respectively.

Ventrites IV with a pair, V–VI each with two pairs of paramedial setae, VII 6-setose apically in male.

Male genitalia (Fig. 5): Median lobe of aedeagus short and stout, moderately sclerotized, with a large sagittal aileron and a large and elongated copulatory piece; ventral margin hardly sinuate, base opening wide, apex broadly rounded; parameres long and broad, but shorter than median lobe, each with four long setae at apex; in dorsal view, apical lobe rounded at apex, nearly as long as wide.

Female: unknown.

##### Etymology.

This species is dedicated to Ms Xiaohong Jin, an active member of Haixia Caving, Guiyang, who found and collected the unique known specimen.

##### Variations.

Both of the paratype specimens have a slightly thinner head than the holotype, and whole body concolorous brown instead of dark brown. Presently, we deal with the differences as individual variations regarding the facts that the similarities of morphological and genital structures, and the caves Dawan Dong and Changtu Dong are close to each other. Molecular analysis would be helpful to clarify their relationship.

##### Distribution.

China (Guizhou). Known from two limestone caves: Dawan Dong and Changtu Dong in the suburb of Guiyang (Fig. 1).

Located in the northern most part of Qingzhen Shi, about 45 km from the main town, Dawan Dong (Fig. 6A) in openings of a cliff of the Maotiaohe valley, on the western side of the river (Fig. 6B). This beautiful cave is 2026 m long, 10–30 m wide and 10–50 m high (Zhao Fei, pers. comm.), with large galleries and several huge chambers (Fig. 6C, D). The single specimen was found running on the ground in a moist and dark area about 500 m from the entrance (Fig. 6 E). Other cave invertebrates found in this cave were woodlice, harvestmen and crickets.

Cave Changtu Dong (Fig. 7) is located about 3.5 km from Dawan Dong in the west, and about half a kilometre from Ximi Village. The cave has two entrances, its length is still unknown. It has been badly impacted and not so pristine as Dawan Dong. Most of the main passage from the smaller entrance is wet and favourable for cave fauna (Fig. 7A, B). The single specimen of *H.jinxiaohongae* gen. nov., sp. nov. was collected together with harvestmen, moths, and millipedes of the genera *Pacidesmus* and *Glyphiulus* along the main passage (Fig. 7C–G).

### Genus *Zhijinaphaenops* Uéno & Ran, 2002

#### ﻿ Zhijinaphaenops zhaofeii sp. nov.

Taxon classificationAnimaliaColeopteraCarabidae

**Figure 8.**
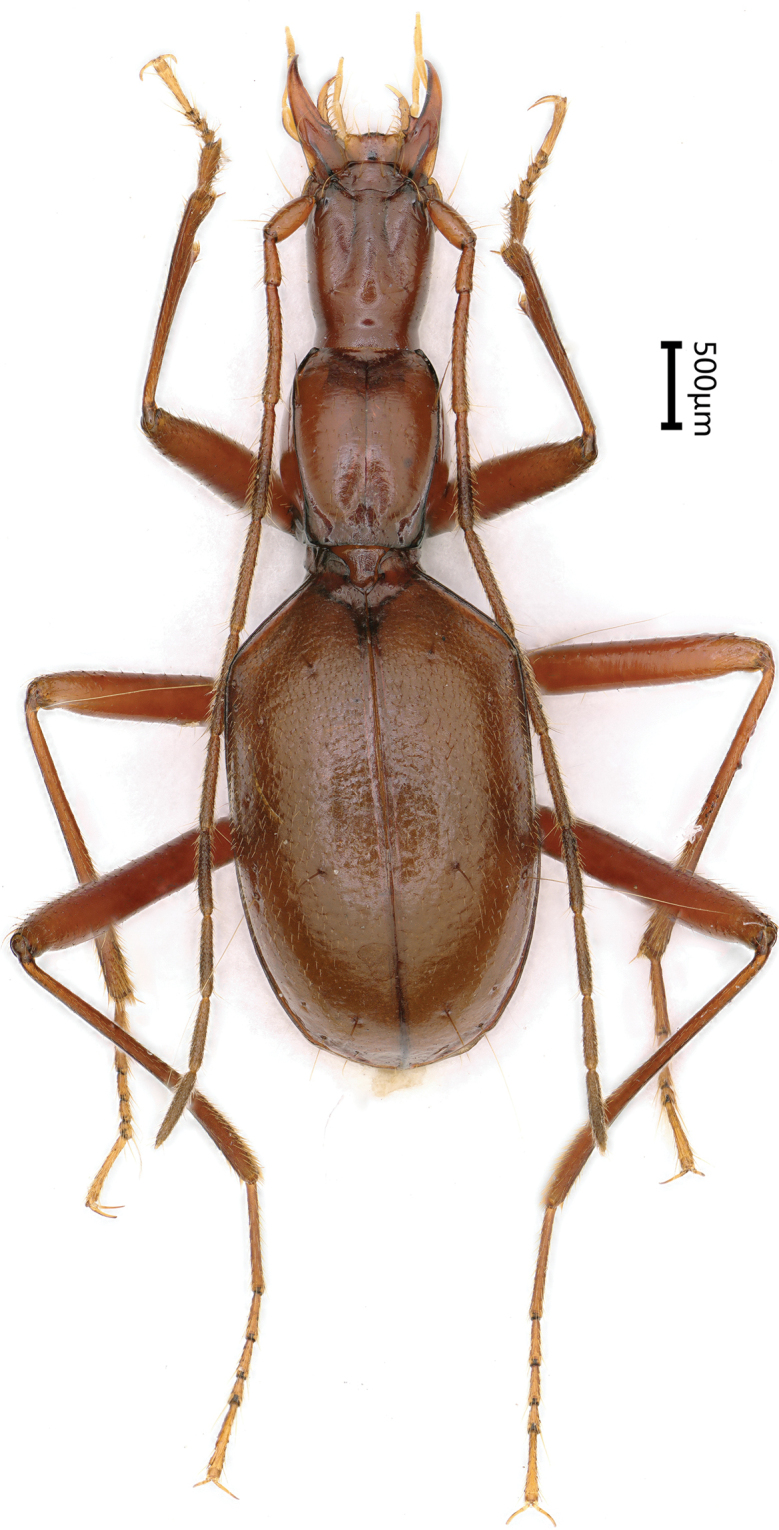
Habitus of *Zhijinaphaenopszhaofeii* sp. nov., holotype, male.

**Figure 9.**
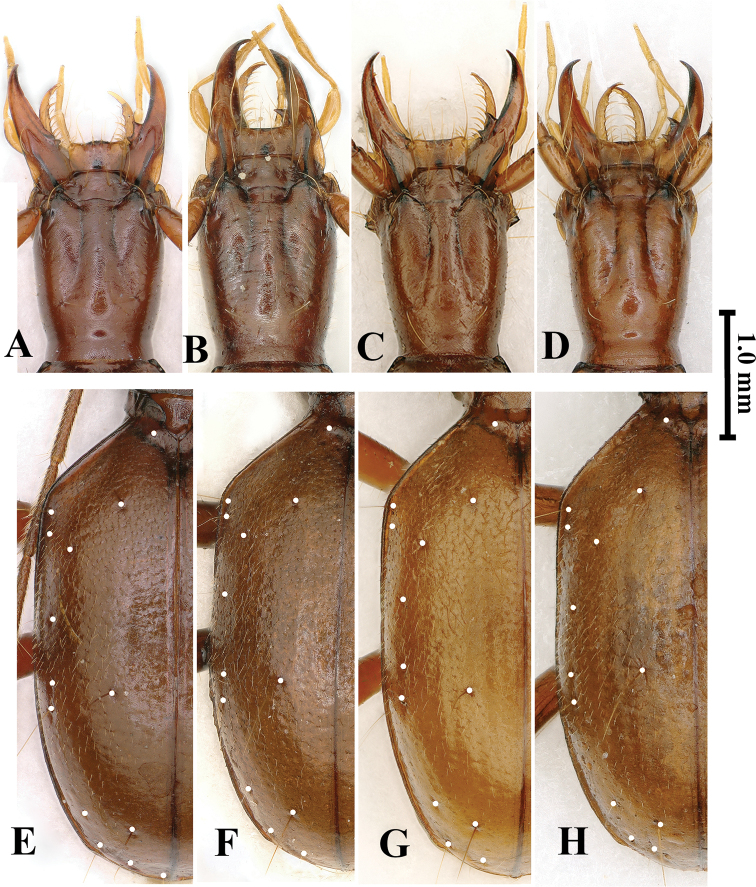
Head (**A–D**) and elytral chaetotaxy (**E–H**) of *Zhijinaphaenops* species **A, E***Z.zhaofeii* sp. nov. **B–D**, **F–H***Z.jingliae***B, F** from Zhangkou Dong in Musan, type locality **C, G** from Mafen Dong **D, H** from Wenquan Dong. Scale bar: 1.0 mm (**A–H**).

**Figure 10.**
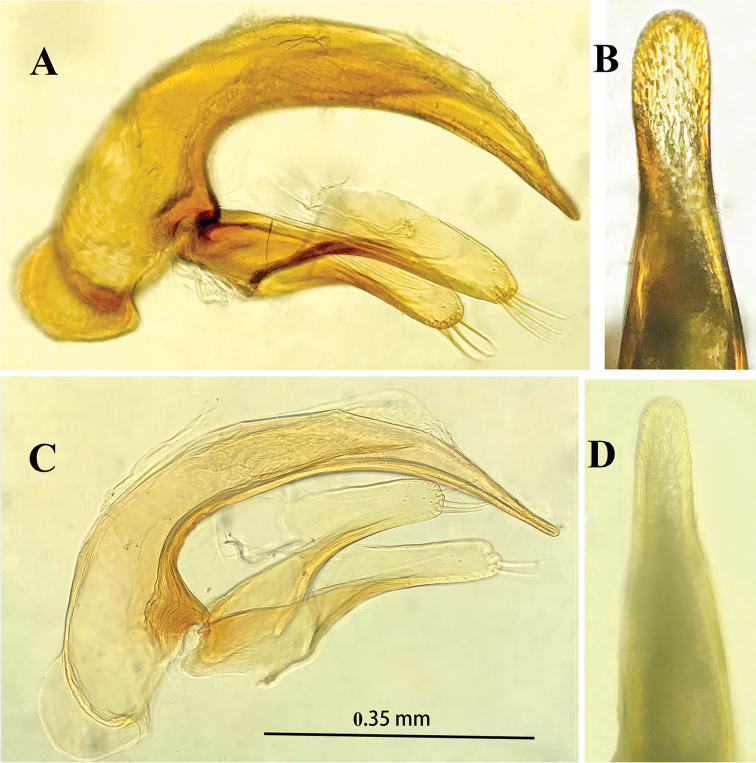
Male genitalia of *Zhijinaphaenops* species, lateral view and apical lobe in dorsal view **A, B***Z.zhaofeii* sp. nov. **C, D***Z.jingliae* Deuve & Tian, 2015. Scale bar for **A–D**.

**Figure 11.**
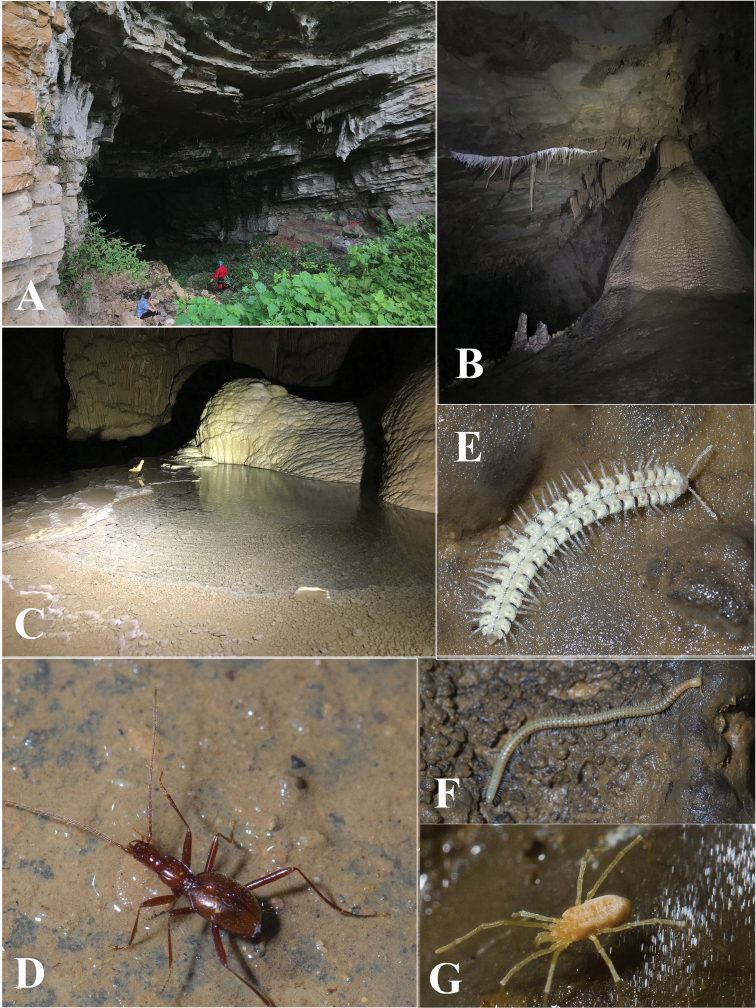
Zhangkou Dong cave, type locality of *Zhijinaphaenopszhaofeii* sp. nov. **A** entrance **B, C** scenes inside the cave **D** a running individual of *Zhijinaphaenopszhaofeii* sp. nov. **E** millipede *Pacidesmus* sp. **F** millipede *Glyphiulus* sp. **G** a mite.

8DF51D98-A57A-5A1D-9344-E131887A1CBA

http://zoobank.org/4A45AFCB-A77F-41B0-A2E8-5E57CDCAFF35

(Chinese name: 赵飞盲步甲) Figures 8 , 9A, E , 10A, B , 11

##### Type material.

***Holotype*** male: Guizhou, Guiyang, Xifeng, Jiuzhuang, Changtu Dong cave (贵州省贵阳市息烽县九庄镇张口洞), 27°11'N, 106°29'E, 1008 m, 2019–VI–08, leg. Jingli Cheng & Mingyi Tian.

##### Diagnosis.

A medium-sized *Zhijinaphaenops* species, body concolorous reddish brown, antennae very long, extending over elytral apices.

##### Description.

Length: 5.8 mm (including mandibles); width: 1.7 mm. Habitus as in Figure 8.

Body reddish brown, palps and tarsi pale. Head covered with sparse and short hairs, whole disc of pronotum covered with dense and long setae; elytra densely pubescent, except glabrous at apical portion; genae, ventral head and prosternum with a few setae; meso- and metasterna and hind coxae dense setose; abdominal ventrites covered with dense and short pubescence.

Microsculptural engraved meshes isodiametric on labrum and base of frons, moderately transverse on vertex and more or less transversally striate on pronotum and elytra. Elytra rather stout, fore body (including mandibles) as long as elytra.

Head (Fig. 9A) moderately elongated, distinctly longer than wide, HLm/HW = 2.25, HLl/HW = 1.66; widest at about middle; sub-parallel-sided, frons and vertex moderately convex; frontal furrows nearly parallel-sided in anterior 2/3, then strongly divergent posteriad, ending before posterior supraorbital pores; anterior supraorbital pores absent; clypeus transverse, 6-setose; labrum transverse, front margin shallowly bisinuate, 6-setose; mandibles thickened, feebly unciform at apex, right mandibular tooth bidentate; labial suture completely disappeared; mentum 2-setose, base widely concave, tooth short, thin and bifid at tip; submentum 9-setose; ligula 2-setose at apex; palpomeres moderately long and slender, glabrous except 2^nd^ labial palpomere bisetose on inner margin, with an additional seta on outer margin near apex; 2^nd^ labial palpomere 1.15 times as long as 3^rd^, whereas 3^rd^ maxillary one 1.20 times longer than 4^th^; suborbital setae close to base of head; antennae slender and elongate, 11^th^ antennomere and part of 10^th^ antennomere extending over elytral apices; scape thick, fusiform, with several rather long setae, pedicle the shortest; antennomeres densely pubescent from pedicle to 11^th^; 3^rd^ antennomeres longest; relative length of each antennomere compared with pedicle in the holotype as follows: 1^st^ (1.27), 2^nd^ (1.00), 3^rd^ (2.57), 4^th^ (2.38), 5^th^ (2.46), 6^th^ (2.22), 7^th^ (2.10), 8^th^ (1.83), 9^th^ (1.75), 10^th^ (1.60) and 11^th^ (1.89).

Prothorax longer than wide, PrL/PrW = 1.14; distinctly wider than pronotum, PrW/PnW = 1.19; shorter than head, PrL/HLm = 0.68, PrL/HLl = 0.92, propleura strongly tumid, widest at about 2/5 from base. Pronotum wider than head, PnW/HW = 1.15; longer than wide, PnL/PnW = 1.35, sides bordered and reflexed throughout, more reflexed near base, widest at about 3/5 from base, more constricted anteriorly, gently contracted backwards, then shallowly sinuate before hind angles; fore angles obtuse, hind angles rectangular; base and front straight, unbordered, and subequal in width; anterior lateral setae at about 1/4 from front, posterior setae absent; disc slightly convex, basal foveae large and deep. Scutellum moderate in size.

Elytra (Fig. 9E) elongated ovate, strongly convex though lateral margins visible throughout from above; much wider than prothorax, EW/PrW = 1.88, much longer than wide, EL/EW = 1.64, widest at about middle; humeral angles broadly rounded; lateral margins finely bordered throughout, smooth, not ciliate; base unbordered, apical striole absent; striae not easily traceable; intervals faintly convex; chaetotaxy (Fig. 9E), anterior discal pore located at about basal 1/6 of elytra, posterior one at about apical 2/5 of elytra; 7^th^ and 8^th^ pores well marked; angulo-apical pore present.

Legs rather long for a *Zhijinaphaenops* species, fore and middle tibiae longitudinally furrowed; the 1^st^ protarsomere in male elongated and widened, denticulate on inner side of apex; 1^st^ tarsomere as long as 2^nd^–4^th^ tarsomeres combined in all legs.

Ventrites IV with 2 pairs, V and VI each with 3 pairs of paramedial setae, VII 6-setose apically.

Male genitalia (Fig. 10A, B): Median lobe of aedeagus short, moderately sclerotized, with a large sagittal aileron and a large and elongated copulatory piece which is about 1/4 of median lobe in length; ventral margin strongly sinuate, then tapering toward apex which is blunt; base opening rather narrow, apical lobe narrow, much longer than wide, broadly rounded at apex; parameres long and broadly widened, but shorter than median lobe, each with 3 long setae at apex.

Female: unknown.

##### Remarks.

Similar to *Zhijinaphaenopsjingliae* Deuve & Tian, 2015, but *Z.zhaofeii* sp. nov. differs in having a wider head, with labrum bisinuate instead of nearly straight, mandibles less hooked at tips and a median lobe with a broader apex in dorsal view.

##### Etymology.

In honor of Mr Fei Zhao, a young active caver in Guiyang.

##### Distribution.

China (Guizhou). Known only from limestone cave Zhangkou Dong, in the suburb of Guiyang (Fig. 1).

Located in the northwestern part of Xifeng County, Zhangkou Dong (Fig. 11) on the southern bank of the Wujiang River. The entrance (Fig. 11A) is close to the main road from Jiuzhuang town to the dock. The passage goes obliquely down to the inner part of the cave, with a small creek inside. It is a rather beautiful cave, but partly damaged by the villagers. The habitat remains favourable for cave animals (Fig. 11B, C). The single beetle was found under a stone in a muddy area about 100 m from the entrance (Fig. 11D). Other cave invertebrates found in this cave were millipedes of *Pacidesmus* and *Glyphiulus* (Fig. 11E, F), a mite (Fig. 11G), moths and crickets.

#### ﻿ Zhijinaphaenops jingliae

Taxon classificationAnimaliaColeoptera Carabidae

Deuve & Tian, 2015

**Figure 12.**
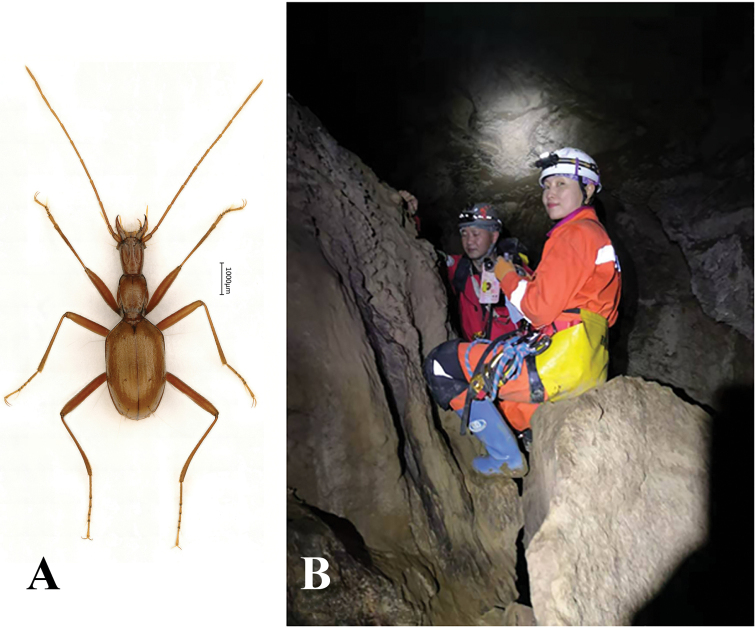
Mafen Dong cave, a new locality of *Zhijinaphaenopsjingliae* Deuve & Tian, 2015 **A** habitus of a male beetle discovered in Mafen Dong **B** place where beetles were discovered.

**Figure 13.**
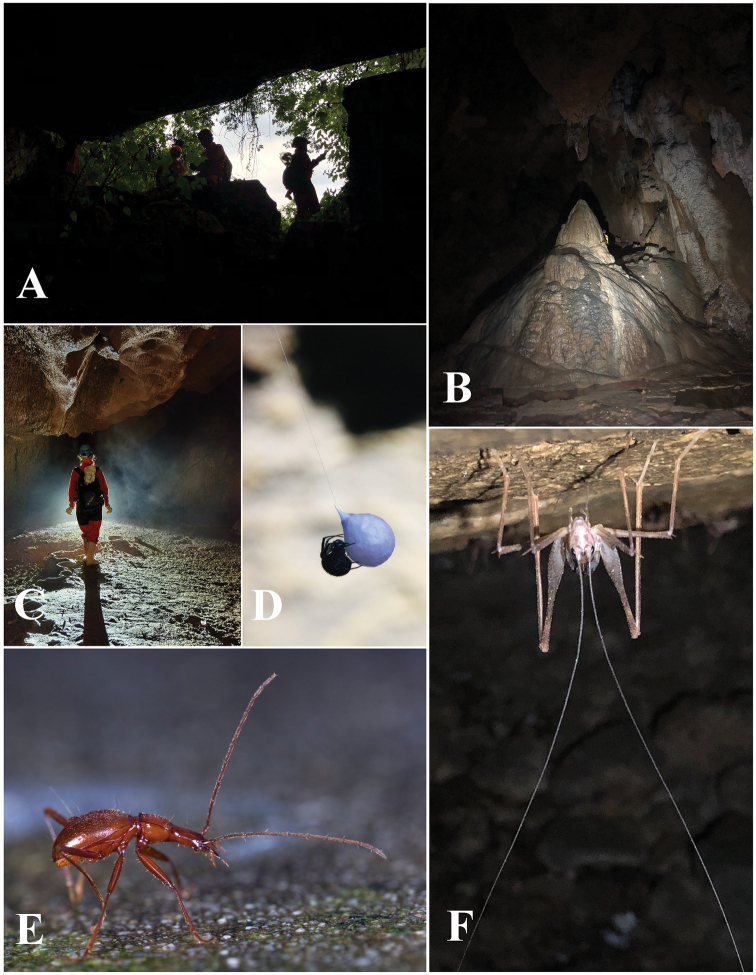
Wenquan Dong cave, a locality of *Zhijinaphaenopsjingliae* Deuve & Tian, 2015 and its sympatric arthropods **A** entrance **B, C** environ inside cave **D** a spider with an egg sac **E** a running individual of *Z.jingliae***F** a *Tachycines* cricket.

83914F6C-DC78-558B-A168-870231E58EEC

(Chinese name: 景丽织盲步甲) Figures 1 , 9B–D, F–H , 10C, D , 12 , 13

Zhijinaphaenops jingliae Deuve & Tian, 2015: 397

##### Material.

2 males & 1 female, Guizhou: Guiyang, Xifeng, Xishan, Shenli, Mafen Dong cave (息烽县西山镇马粪洞), 27°04'N, 106°37'E, 1157 m, 2020–II–29, leg. Guangyuan Cheng; 1 male, Guizhou, Guiyang, Xifeng, Xinlong, Wenquan Dong (息烽县温泉镇温泉洞), 27°11'N, 106°49'E, 916 m, 2020–VII–18, leg. Jingli Cheng.

##### Diagnosis.

A medium-sized *Zhijinaphaenops* species, wholly brown and pubescent, head narrow, with thin and very long antennae which extend beyond of elytral apices. Habitus as in Figure 12 A.

##### Remarks.

*Zhijinaphaenopsjingliae* was recorded from Zhangkou Dong cave near Musa village, Shidong Zhen, Xifeng County (Deuve and Tian 2015). In 2020, this species was found in two caves of the same county, Mafen Dong (Xishan Zhen) and Wenquan Dong (Wenquan Zhen). Morphological characteristics including male genitalia of individuals from the above two caves are identical to the holotype specimen (Figs 9B–D, F–H, 10C, D).

##### Distribution.

China (Guizhou). Known from three limestone caves in Xifeng County, northern Guiyang (Fig. 1).

Mafen Dong cave (Fig. 12B) is about 600 m from Mafen village, only 5 km away Zhangkou Dong cave in the east. The individuals of *Z.jingliae* were found running on the ground in a dark area not far from the entrance.

Wenquan Dong cave is near Xinglong village, about 30 km from Zhangkou Dong. It opens via quite large entrance in a small hill of a valley and is surrounded by bushes, (Fig. 13A). There are two large chambers inside and a main passage about two hundred meters long. There is a pool at the bottom of the first chamber. A large part of the cave is moist and maintains good conditions for cave animals (Fig. 13B, C). Apart from the single beetle of *Zhijinaphaenopsjingliae*, other cave arthropod animals found in this cave were spiders and crickets (Fig. 13D–F).

### Genus *Sinaphaenops* Uéno & Wang, 1991

#### ﻿ Sinaphaenops chengguangyuani

Taxon classificationAnimaliaColeopteraCarabidae

Ma et al. 2020

**Figure 14.**
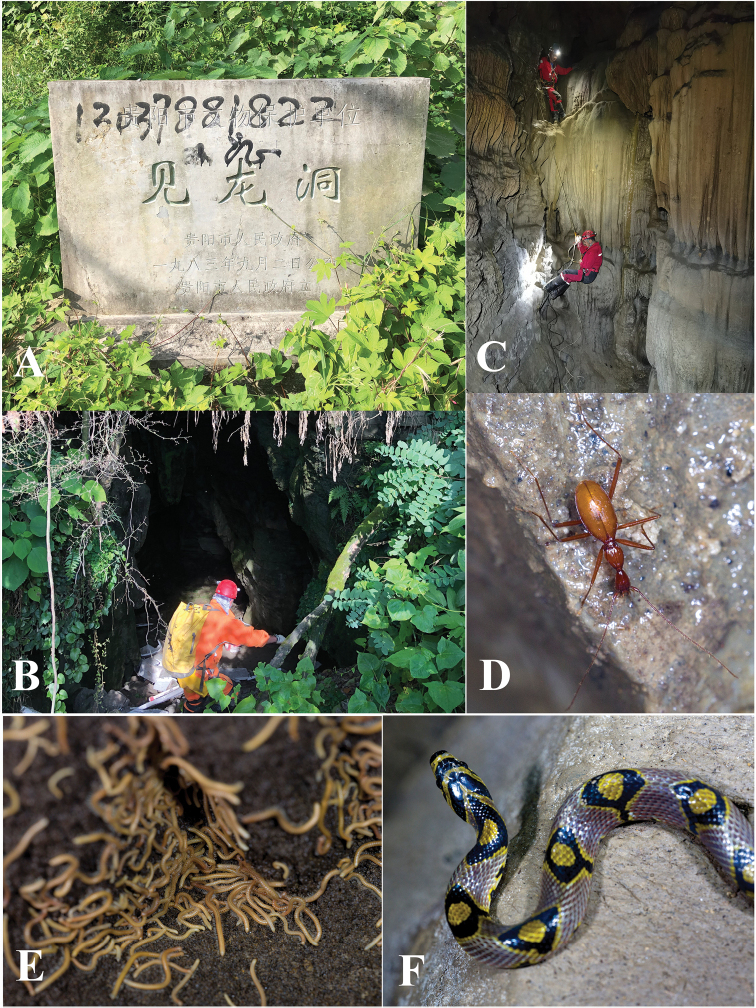
Jianlong Dong cave, a new locality of *Sinaphaenopschengguangyuani*Ma et al. 2020**A** a monument indicating that the cave is under protection **B** entrance **C** stalagmites in the inner chamber **D** a running beetle of *S.chengguangyuani***E** millipedes of *Glyphiulus* sp. **F** a snake *Elaphemandarina* (Cantor, 1842).

**Figure 15.**
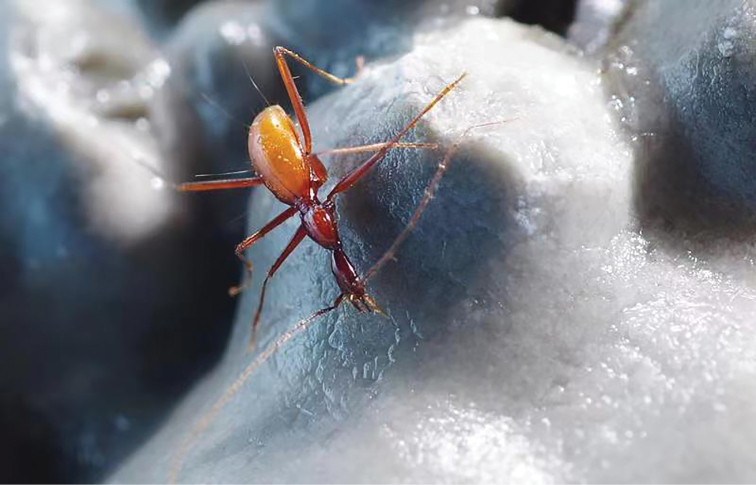
A running individual of *Sinaphaenopschengguangyuani*Ma et al. 2020 inside Duocai Dong cave.

21A5738F-BD85-57AA-B137-3F1ADA3BBEE1

(Chinese name: 程广源华盲步甲) Figures 1 , 14 , 15

Sinaphaenops chengguangyuani Ma et al. 2020: 582

##### Material.

1 male & 4 females, Guizhou, Guiyang, Nanming, Yunguan, Jianlonglu, Jianlong Dong cave (贵州省贵阳市南明区见龙洞), 26°32'N, 106°46'E, 1094 m, 2020–VI–26, leg. Guangyuan Cheng, Xiaohong Jin, Chenggang Wang, Yi Zhao & Mingyi Tian; 1 female & 1 male, Guizhou, Qiannan Miao & Buyi Autonomous Prefecture, Longli, Duocai Dong (贵州省黔南布依族苗族自治州龙里县多彩洞), 26°34‘N, 106°59‘E, 1555 m, 2021–VIII–14, leg. Guangyuan Cheng.

##### Distribution.

China (Guizhou). Known from three limestone caves in Guiyang Shi and Longli County, Qiannan Buyi & Miao Autonomous Prefecture (Fig. 1).

This species was described from Shuijing Dong cave, Longli County, Qiannan Buyi & Miao Autonomous Prefecture (Ma et al. 2020). Then it was found in Jianlong Dong, Guiyang and Duocai Dong, Longli.

Jianlong Dong cave (Fig. 14) is located at the eastern suburb of Guiyang, about 40 km from Shuijing Dong, the type locality of *S.chengguangyuani*. This cave is a municipal protected area because of the engraved inscriptions on the precipices inside dating from the Ming Dynasty, over 400 years ago (Fig. 14A). The rather large entrance is just on the side of the road Jianlonglu (Fig. 14B). The cave is about 100 m long, with two chambers inside. The first half part is rather dry, then becomes wet in the inner part (Fig. 14C). All of the beetle individuals were collected in the inner part, running on the ground or on the wall. Other cave animals observed in this cave were millipedes (*Glyphiulus* sp., Fig. 14E), a snake (*Elaphemandarina* Cantor, 1842; Fig. 14F), crickets and moths.

Duocai Dong is a stalagtite-rich cave, about 5 km from Shuijing Dong in straight line. The two individuals were collected in dark zone, running on the wall (Fig. 15).

## Supplementary Material

XML Treatment for Haixiaphaenops

XML Treatment for Haixiaphaenops jinxiaohongae

XML Treatment for Zhijinaphaenops zhaofeii

XML Treatment for Zhijinaphaenops jingliae

XML Treatment for Sinaphaenops chengguangyuani
